# Laparoscopic Vessel Endometriosis Resection Surgery: A Case Report and Review of Literature

**DOI:** 10.1155/2019/1375208

**Published:** 2019-12-12

**Authors:** Shadi Rezai, Richard A. Giovane, Tiffanie Mann, Ninad M. Patil, Elise Bardawil, Cassandra E. Henderson, Xiaoming Guan

**Affiliations:** ^1^Division of Minimally Invasive Gynecologic Surgery, Department of Obstetrics and Gynecology, Baylor College of Medicine, 6651 Main Street, 10^th^ Floor, Houston, TX 77030, USA; ^2^University of Alabama, Department of Family Medicine, 801 Campus Drive, Tuscaloosa, AL 35487, USA; ^3^Department of Pathology & Immunology, Baylor College of Medicine, 6651 Main Street, Suite F0455.10, Houston, TX 77030, USA; ^4^Maternal and Fetal Medicine, Department of Obstetrics and Gynecology, Lincoln Medical and Mental Health Center, 234 East 149^th^ Street, Bronx, NY 10451, USA

## Abstract

**Background:**

Endometriosis usually occurs in the pelvis and often involves the ovaries, the uterosacral and broad ligaments, and the pelvic peritoneum. In rare instances, it can occur in the vasculature of the pelvis. Patients with endometriosis present with abnormal pain, menstrual cycle disruption and infertility. Management of endometriosis is usually surgical with excision of the tissue via laparoscopic means.

**Case:**

A 42-year-old Gravida 5, Para 2-0-3-2 patient with a 22 year history of endometriosis, who had had multiple laparoscopic endometriosis resections, total abdominal hysterectomy, and an exploratory laparotomy with bilateral salpingo-oophorectomy, presented with left pelvic pain when standing, dyspareunia, and a 3.7 cm cyst on ultrasound. The patient underwent laparoscopic vessel endometriosis resection and excision of endometriotic nodules from external iliac vessels. Final pathology report showed evidence of old endometriosis in all locations. On interval follow-up, the patient reported sustained relief from pain.

**Conclusion:**

Complete resection of endometriosis from large vessels can be successfully achieved laparoscopically by a well-experienced surgeon with delicate, proper techniques.

## 1. Background

Endometriosis is defined as the presence of endometrial tissue, including glands and/or stroma, occurring anywhere outside of the uterine cavity [1]. The ectopic tissue embeds and infiltrates itself into nearby structures and responds cyclically to sex hormones, eliciting an inflammatory response. Over time, there may be fibrosis in the surrounding tissue. Occasionally, the endometriotic tissue may be replaced by collagen and scar tissue, referred to as “burnt-out endometriosis” [1, 2]. While the precise etiology of endometriosis is unclear, retrograde menstruation and/or metaplasia of the peritoneal lining are postulated mechanisms in most cases. It remains one of the most common gynecological disorders of the female reproductive tract [2]. In symptomatic women, it classically presents with chronic pelvic pain and infertility [2]. Endometriosis is graded in severity using surgical staging: from stage I correlated with minimal disease, to stage 4 signifying severe or complex disease [5].

While endometriotic lesions usually develop within the pelvic cavity, affecting the ovaries and the ligamentous structures between the uterus and the peritoneum, it is not uncommon to identify lesions beyond these structures [1]. Vessel endometriosis is a rare phenomenon of severe endometriosis whereby fibrotic endometriotic nodules appear in the vasculature of the pelvis [6]. Endometriosis has a preponderance for recurrence, as retreatment is evidenced in many cases as the primary reasoning for surgical procedures such as laparoscopy [8]. Additionally, rates of retreatment appear to be significantly reduced following excision and removal via laparoscopy [8]. Hence, a more definitive treatment must be used to decrease recurrence. Laparoscopy is a less invasive surgical approach utilized to eradicate endometriotic lesions [7, 8]. It also drastically reduces the amount of time necessary for adequate postoperative healing and recovery [9, 10]. The objective of our review of the literature is to demonstrate the benefits of laparoscopic resection in a novel case of complex endometriosis affecting the external iliac vasculature, ureter, bowel and rectum.

## 2. Presentation of the Case

The patient, is a 42-year-old white female Gravida 5, Para 2-0-3-2, with a past medical history of endometriosis, diagnosed since age 19.

The patient presented to our clinic with severe pelvic pain, particularly on the left side, with associated dysmenorrhea, dyschezia and dyspareunia. The patient reported that this pain was constant and had been ongoing for a few months. Pelvic pain was exacerbated upon standing; there were no alleviating factors. Her physical exam revealed positive pelvic pain preponderant on the left side. No nodularity was palpable on rectal examination. The rest of the physical exam was unremarkable. Based on the patient's presentation and physical exam, it was decided to do a pelvic ultrasound. The ultrasound showed a 3.7 cm septated cystic mass in the left adnexa (ovarian remnant cyst) on ultrasound.

Upon further chart review, the patient had multiple previous surgeries. These include two cesarean deliveries, two laparoscopic endometriosis resections and fulguration of endometriotic implants. The patient also had a laparoscopic left ovarian cystectomy for a 10 cm endometrioma (which recurred as a 5 cm endometrioma within 6 months), two exploratory laparotomies, including a total abdominal hysterectomy (TAH) and an exploratory laparotomy with bilateral salpingo-oophorectomy (BSO).

**Figure 1:**
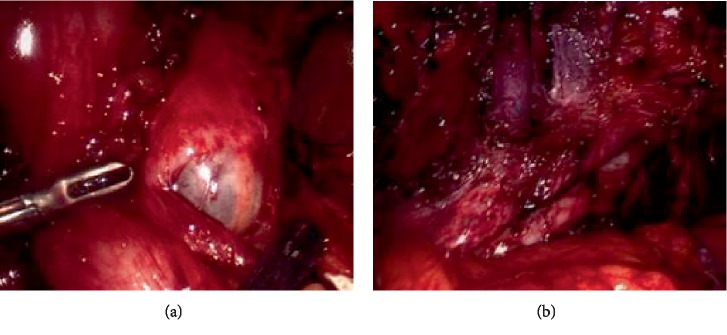
Intraoperative images: (a) left pelvic cyst (a 4 cm ovarian remnant cyst) was identified and the entire ovary was removed; (b) endometriotic nodules and endometriosis on left external iliac artery and vein.

**Figure 2:**
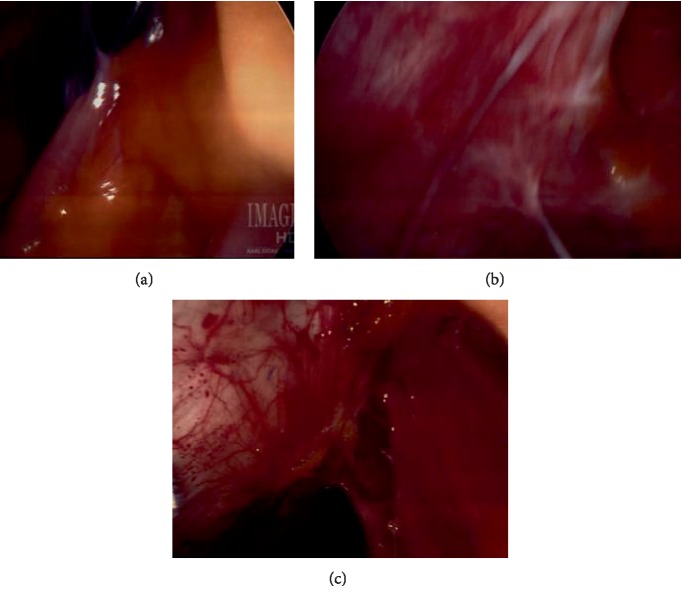
Intraoperative images: (a) adhesion of bowel and omentum to anterior abdominal wall; (b) white patches of endometriotic implants; (c) endometriotic implants on the large bowel (left), adhesions of omentum to large bowel (right).

The patient underwent laparoscopic resection of endometriotic implants and nodules from left external iliac artery and vein (Figure 1(b)), bowel resection, ureteral lysis, left pelvic cyst (ovarian remnant cyst) removal (Figure 1(a)), left pelvic nodule resection, lysis of adhesions (Figure 2(a)), cystoscopy and stent placement for management of endometriosis (Figure 2(b)) and pelvic pain.

*Operative Technique (17):* The Enseal device was used to lyse omental adhesions and cold scissors were used to lyse bowel adhesions. A 4 cm ovarian remnant was identified and the entire ovary was removed (Figure 1(a)). The left ureter was completely dissected out in order to completely remove the left ovarian remnant cyst. The endo-GIA stapler was used to transect the bowel. Bowel endometriosis was resected with the TA stapler though the 5 cm umbilical incision (Figure 2(c)). A rigid nodule was detected at the left external iliac vessels and was dissected out and removed using sharp and blunt dissection. The harmonic was used to transect the fibrotic tissues of the endometriosis nodule. The ureteral endometriosis was also transected. A left ureteral stent was placed and left in for 4 weeks.

**Figure 3:**
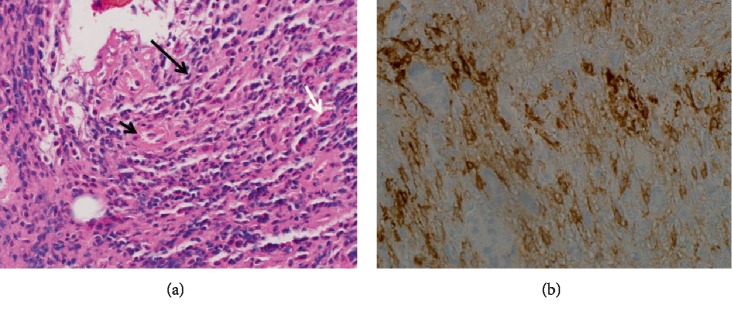
Pathology slides: left external iliac nodule biopsy: (a) endometrial-type stroma (long black arrow) and capillary (short black arrow) with hemosiderin-laden macrophage (white arrow). (H&E stain); (b) endometrial-type stroma is positive for CD10 (CD10 immunohistochemical stain).

The final pathology report (Figures 3(a) and 3(b)) confirmed evidence of burnt-out endometriosis in all locations including bowel and external iliac nodule. In the left external iliac vessel, residual endometrial-type stroma, vasculature, and hemosiderotic macrophages were seen on H&E stain (Figure 3(a)), and highlighted by CD10 immunohistochemical stain (Figure 3(b)).

On interval follow-up, the patient reported sustained relief from pain, last recorded at 9 months intraoperatively.

## 3. Discussion

Review of the literature shows that complete resection of endometriotic lesions from the affected vasculature can be utilized to successfully treat chronic pelvic pain secondary to severe endometriosis [11, 12]. Pelvic pain usually returns within one year of treatment in patients receiving medical therapy such as GnRH agonists; conversely, Sutton et al. showed that 90% of patients receiving laparoscopy for endometriosis were symptom-free at one year [8]. While endometriosis is a common finding in chronic pelvic pain and a known factor affecting fecundity, a small subset of women have deeply infiltrating endometriosis, affecting distant organs and structures [6]. Such patients with severe disease are often plagued with high rates of recurrence as it can be difficult to (1) identify all lesions during one laparoscopic procedure and/or (2) resect and completely remove endometriotic implants involving more delicate structures (i.e., bowel, ureter and vessels), requiring extreme skill and anatomical knowledge [13, 14]. In this case, the patient had a history of chronic, debilitating endometriosis despite numerous surgeries designed to substantially reduce or eliminate symptoms.

Overall, laparoscopic procedures are associated with a shorter hospital stay, faster healing time, and decreased morbidity. However, morbidity may be increased in patients who undergo repeat procedures when prior surgical treatment has been unsuccessful [12, 15–17]. In our case, the patient did not report complete pain resolution until after complete resection of the fibrotic nodules lining the external iliac artery and vein. In patients with known severe disease who report persistent pain despite multiple medical and surgical therapies, it may prove worthwhile to thoroughly investigate less commonly affected structures to identify fibrotic nodules and other pathologic changes associated with endometriosis.

## 4. Conclusion

Complete resection of endometriosis, including from large vessels may be the key to successful surgical treatment. With sufficient experience, knowledge of anatomy, and precise dissection technique, endometriomas can be safely removed from large vessels [17].
